# A Novel Curcumin Analog (H-4073) Enhances the Therapeutic Efficacy of Cisplatin Treatment in Head and Neck Cancer

**DOI:** 10.1371/journal.pone.0093208

**Published:** 2014-03-27

**Authors:** Bhavna Kumar, Arti Yadav, Kalman Hideg, Periannan Kuppusamy, Theodoros N. Teknos, Pawan Kumar

**Affiliations:** 1 Department of Otolaryngology-Head and Neck Surgery, The Ohio State University, Columbus, Ohio, United States of America; 2 The Ohio State University Comprehensive Cancer Center, The Ohio State University, Columbus, Ohio, United States of America; 3 Institute of Organic and Medicinal Chemistry, University of Pécs, Pécs, Hungary; 4 Geisel School of Medicine, Dartmouth, Hanover, New Hampshire, United States of America; Winship Cancer Institute of Emory University, United States of America

## Abstract

Chemotherapy constitutes the standard modality of treatment for localized head and neck squamous cell carcinomas (HNSCC). However, many patients fail to respond and relapse after this treatments due to the acquisition of chemo-resistance. Therefore, there is an urgent need to develop novel drugs that could reverse the resistant phenotype. Curcumin, the constituent of the spice turmeric has been shown to have anti-inflammatory, anti-oxidant and anti-proliferative properties in several tumor types. However, use of curcumin has been limited due to its poor bio-absorption. Recently, a novel class of curcumin analogs, based on diarylidenylpiperidones (DAP), has been developed by incorporating a piperidone link to the beta-diketone structure and fluoro substitutions on the phenyl groups. In this study, we evaluated the effectiveness of H-4073, a parafluorinated variant of DAP, using both *in vitro* and *in vivo* head and neck cancer models. Our results demonstrate that H-4073 is a potent anti-tumor agent and it significantly inhibited cell proliferation in all the HNSCC cell lines tested in a dose-dependent manner. In addition, pretreatment of cisplatin-resistant HNSCC cell lines with H-4073 significantly reversed the chemo-resistance as observed by cell viability assay (MTT), apoptosis assay (Annexin V binding) and cleaved caspase-3 (Western blot). H-4073 mediated its anti-tumor effects by inhibiting JAK/STAT3, FAK, Akt and VEGF signaling pathways that play important roles in cell proliferation, migration, survival and angiogenesis. In the SCID mouse xenograft model, H-4073 significantly enhanced the anti-tumor and anti-angiogenesis effects of cisplatin, with no added systemic toxicity. Interestingly, H-4073 inhibited tumor angiogenesis by blocking VEGF production by tumor cells as well as directly inhibiting endothelial cell function. Taken together, our results suggest that H-4073 is a potent anti-tumor agent and it can be used to overcome chemotherapy resistance in HNSCC.

## Introduction

Head and neck squamous cell carcinoma (HNSCC) is one of the most prevalent cancer worldwide with more than 600,000 cases are diagnosed every year [1]. Tobacco usage and alcohol consumption have been known to be the strongest risk factors for the development of this disease [2]. However, it is now being recognized that human papillomavirus (HPV) can also play a role in the development of a subset of head and neck cancers [3]. Most of the HNSCC are diagnosed in advanced stages and the outcome of these patients is often poor [4]. Cisplatin is one of the commonly used chemotherapeutic agents for the treatment of head and neck cancers [5]. Cisplatin is an inorganic platinum agent, which can bind to DNA, inducing intrastrand and interstrand DNA cross-links, as well as DNA-protein cross-links. These cross-links result in apoptosis and cell-growth inhibition [6]. However, many patients develop resistance to cisplatin treatment leading to the treatment failure [7]. Therefore, there is a need to develop novel therapeutic strategies that are more effective and have fewer side effects than currently used treatment regimens.

The signal transducers and activators of transcription (STATs) are a family of signaling proteins that are activated by cytokines and growth factors [8]. To date, 7 members of the STAT family have been identified in mammals. In normal cells, STAT proteins get transiently activated by phosphorylation and play a role in cytokine and growth factor-mediated responses like cell growth, survival and inflammation [9], [10], [11]. However, it has been observed in numerous studies that STAT3 is constitutively active in a variety of human solid tumors including lung cancer [12], prostate cancer [13], breast cancer [14] and in more than 95% of head and neck cancers [10]. Dysregulation and constitutive activation of STAT3 stimulates cancer cell growth and contributes to tumor development and progression by upregulation of STAT3 target genes including Bcl-2, c-myc, cyclin D1 and VEGF, which can enhance cell survival, proliferation and promote angiogenesis [15], [16], [17]. Increased STAT3 expression has been linked to poor prognosis in patients with gastric, colorectal cancers, cervical squamous cell carcinoma and gliomas [18], [19], [20]. In addition, STAT3 overexpression and signaling have been found to be associated with cisplatin resistance in HNSCC [21], [22]. Therefore, intense efforts have focused on targeting STAT3 signaling using novel therapeutic approaches which could induce apoptosis and sensitize tumors to chemotherapy [23].

Recently, a novel class of diarylidenylpiperidone (DAP) compounds has been synthesized by incorporating a piperidone ring in the beta-diketone backbone structure of curcumin [24]. These compounds showed substantially higher anti-cancer activity than curcumin in an ovarian cancer model [25]. It was also found that these compounds inhibited the activation of STAT3. The aim of this study was to evaluate the efficacy of one of the diarylidenylpiperidone (DAP) compound, H-4073 either as a single agent or in combination with cisplatin, both *in vitro* and *in vivo*. We show that H-4073, as a single agent, was effective in inhibiting tumor cell proliferation and inducing apoptosis. Furthermore, H-4073 mediated inhibition of STAT3, focal adhesion kinase (FAK), Akt and vascular endothelial cell growth factor (VEGF) signaling pathways and was able to sensitize tumor cells to the effects of cisplatin, resulting in inhibition of migration and apoptosis *in vitro* as well as reduction in tumor volume *in vivo*. Our data support the therapeutic use of H-4073 in combination with cisplatin in the treatment of head and neck cancer.

## Materials and Methods

### Ethics Statement

All animal work was approved by the Ohio State University IACUC Animal ethic committee and conducted according to their guidelines (Animal Welfare Assurance Number A3261-01).

### Cell Lines and Reagents

UM-SCC-74A, UM-SCC-1, UM-SCC-74B, UM-SCC-38 and UM-SCC-47 were obtained from the laboratory of Dr. Thomas E. Carey at the University of Michigan [26]. CAL27 was obtained from ATCC (Manassas, VA). Cisplatin resistant cell line (CAL27-CisR) was generated from its parental CAL27 cell line (ATCC) as previously described [27]. The identity of all cell lines was authenticated by STR genotyping (AmpFlSTR Identifiler PCR Amplification Kit, Applied Biosystems, Carlsbad, CA). All the head and neck cancer cell lines were cultured in Dulbecco's modified Eagle medium (DMEM, Invitrogen, Carlsbad, CA), 10% fetal bovine serum, 1% penicillin/streptomycin and 1% non-essential amino acids (Invitrogen). H-4073 was synthesized as described previously [24]. The stock compound was freshly dissolved in DMSO for *in vitro* work. For *in vivo* work, H-4073 was mixed with animal feed (50 ppm dose by Harlan Tekland). Antibodies against pSTAT3 (Tyr705), pAkt (Ser473), pp38 (Thr180/Tyr182), pERK1/2 (Thr202/Tyr204) and GAPDH were obtained from Cell Signaling Technology (Danvers, MA) and pFAK (Tyr397) antibody was from Abcam (Cambridge, MA). CD31 antibody was from Dianova (Hamburg, Germany) and p21 antibody was obtained from Calbiochem (EMD Millipore, Billerica, MA).

### Cellular Uptake

To determine the uptake of H-4073 by HNSCC cells *in vitro*, UM-SCC-74A cells were grown in 10-cm dishes and treated with10 µM of H-4073 or 100 µM curcumin. After 1 hour of incubation, the cells were trypsinized, counted, and washed with PBS. The cell pellet was allowed to dry, resuspended in methanol and sonicated for 15 min. The sonicate was centrifuged at 10000 rpm for 5 minutes at 4°C. The supernatant was diluted with an equal volume of methanol and measured with a UV/Vis spectrophotometer (λ = 328 nm, ε = 25000 M^−1^ cm^−1^).

### Cell Proliferation Assay

The sensitivity of cells to cisplatin and H-4073 was measured using the MTT-based colorimetric cell proliferation kit (Roche Applied Science, Mannheim, Germany) [27]. Briefly, 5000 cells/well were plated in a 96 well plate. The next day, cells were treated with different concentrations of H-4073, cisplatin, or a combination of both. After 72 hours, 10 µl/well of MTT solution was added to each well and further incubated for 4 hours at 37°C. The formazan crystals formed in the wells were solubilized by adding solubilization solution and incubating the plates at 37°C overnight. The plates were read at 590 nm on a Spectramax 190 plate reader (Molecular Devices Inc., Sunnyvale CA). The percentage cell growth inhibition for each treatment group was calculated by adjusting the untreated control group to 100%. Data were analyzed using GraphPad Prism software (GraphPad Sofware, Inc., San Diego, CA) and the dose response curves were used to calculate the concentration of H-4073 or cisplatin resulting in 50% inhibition of cell proliferation (IC50) using a four parametric logistical model. All experiments were repeated at least 3 times.

For drug combination studies, the synergistic effect was assessed by the combination index (CI), according to the method of Chou and Talalay wherein synergism is defined as CI<1, while antagonism is CI>1, and an additive effect is considered as CI = 1 [28]. The CI values were calculated using CompuSyn software (ComboSyn, Inc., Paramus, NJ).

### Colony formation assay

Tumor cells were plated in 6 cm dishes and treated with cisplatin, H-4073 or a combination of both. After 48 hours of treatment, 4×10^3^ viable cells from each group were plated in 6 cm dishes and cultured for additional 10 days. The colonies were fixed with methanol and stained with crystal violet. Photomicrographs were taken and the number of colonies was counted by Alpha Innotech imaging software (San Leandro, CA).

### Apoptosis Assay

Cells were plated in 6-cm dishes and treated for 24 hours. Cell culture supernatant and cells were collected 48 hours after treatment. Cells were stained with Annexin V 488 (Life Technologies, Grand Island, NY) and propidium iodide (Sigma, St. Louis, MO) and analyzed by flow cytometry [27].

### Immunoblotting

Cell lysates were run on Novex Bis-Tris gel (Invitrogen) under reducing conditions, blotted onto PVDF membranes (GE Healthcare Life Sciences/Amersham, Piscataway, NJ), probed with primary antibodies, then rinsed and incubated with sheep anti-mouse or donkey anti-rabbit conjugated with horseradish peroxidase (GE Healthcare). The membranes were visualized using the ECL Plus Kit (GE Healthcare).

### Migration Assay

The effect on cell migration upon treatment was measured using the xCELLigence RTCA DP Instrument (Roche Applied Science, Mannheim, Germany) [29]. Briefly, cells were grown in serum-free medium for 24 hours. The bottom chamber of the CIM-plate 16 was filled with 160 µl of complete medium. The bottom and top chambers were snapped together. Serum-free medium was placed in the top chamber and incubated for 1 hour in the CO_2_ incubator at 37°C. Cells were trypsinized, pelleted and resuspended so that 80,000 cells were added to each well of the top chamber in serum free medium containing cisplatin, H-4073 or a combination of both. The CIM-Plate 16 was placed in the RTCA DP station and migration was monitored for 24 hours.

### In vivo Studies

For anti-tumor efficacy studies, nude athymic mice bearing tumor xenografts were used. All animal experiments were performed under the guidelines of The Ohio State University Committee for Use and Care of Animals. Tumor cells (1×10^6^) were mixed with 100 µl of Matrigel and injected subcutaneously in the flank area of the mice [30]. After 8 days, mice were stratified into different groups (n = 5), so that the mean tumor volume in each group was comparable. Animals were treated with H-4073 by mixing it with feed (50 ppm dose, Harlan Teklad) starting at day 8. Animals were treated with cisplatin (5 mg/kg) at days 8, 11, 14, 17, 21, 24, and 28 via intraperitoneal injections. Tumor volume measurements [volume (mm^3^) = L×W^2^/2 (length L, mm; width W, mm)] began on day 6 and continued twice a week until the end of the study. After 30 days, primary tumors were carefully removed, photographed, and analyzed for pSTAT3, TUNEL-positive cells and tumor angiogenesis.

### Immunohistochemistry

The xenograft tumor tissues were fixed in 4% paraformaldehyde overnight and paraffin embedded. Tissue sections were deparffinized, pretreated with antigen retrieval buffer (EDTA, pH 8.0; pSTAT3) (Citrate, pH 6.0; CD31) [27]. Endogenous peroxidase and non-specific binding sites were blocked and the sections were incubated with pSTAT3 (Tyr 705) or CD31. The primary antibody binding was detected using biotinylated goat anti-rabbit IgG (Vector Laboratories, Burlingame, CA) or biotinylated goat anti-rat IgG (BD Biosciences, San Jose, CA). The slides were then incubated with avidin-biotin complex (Vector Laboratories). The reaction sites were visualized using 3,3′-diaminobenzidine (DAB; Sigma Aldrich, St. Louis, MO). The sections were counterstained with hematoxylin, dehydrated and mounted with Permount.

The ApopTag Peroxidase In Situ Apoptosis Detection Kit (EMD Millipore, Billerica, MA) was used to detect apoptotic cells by terminal deoxynucleotidyl transferase dUTP nick end labeling (TUNEL) staining in the xenograft tumor sections according to the manufacturer's protocol. Briefly, sections were deparaffinized, rehydrated and incubated with Proteinase K for 15 minutes at room temperature and then washed. The endogenous peroxidase activity was quenched and after washing the sections were incubated with working concentration of terminal deoxynucleotidyl transferase (TdT) at 37°C for 1 hour. The sections were then washed and incubated with anti-digoxigenin conjugate (peroxidase) at room temperature. The signal was visualized with DAB as chromogen.

### Statistical Analysis

Data from all the experiments are expressed as mean ± SEM from a minimum of 3 independent experiments. The statistical significance of the results was evaluated by two-way analysis of variance or Student's t test (wherever applicable) and a p value of <0.05 was considered significant. IC_50_ values for H-4073 and cisplatin for tumor cell proliferation inhibition was calculated using GraphPad Prism software (GraphPad Sofware, Inc., San Diego, CA) and combination index (CI) was calculated using CompuSyn software (ComboSyn, Inc., Paramus, NJ).

## Results

### H-4073 is a potent inhibitor of HNSCC proliferation

**Figure 1:**
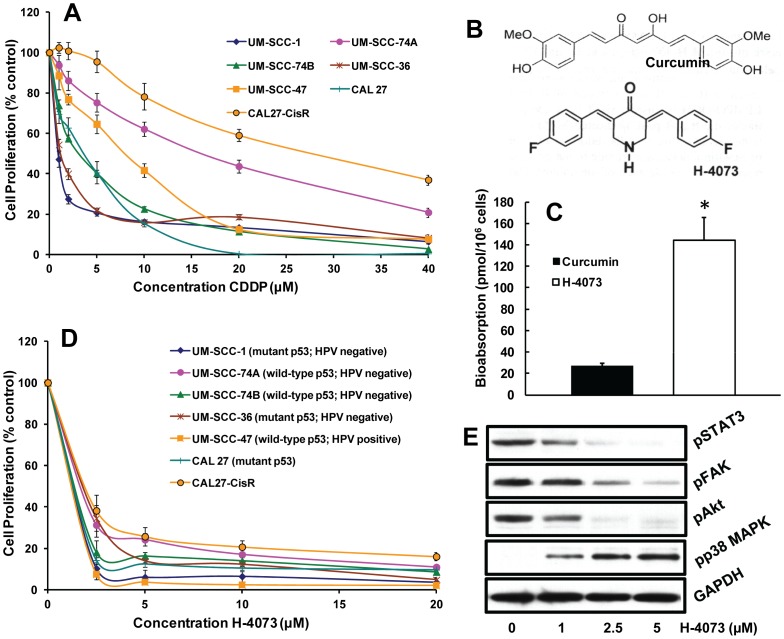
H-4073 inhibits STAT3 activation and head and neck cancer cell proliferation in a dose dependent manner. ***A:*** HNSCC cell lines were treated with different concentrations of cisplatin (CDDP) and cell proliferation was assessed by MTT assay. ***B:*** Chemical structures of curcumin and H-4073. ***C:*** UM-SCC-74A cells were cultured in 10-cm culture dishes and treated with curcumin or H-4073 for 1 hour. Cellular uptake of curcumin and H-4073 was measured with a UV/Vis spectrophotometer. ***D:*** HNSCC cell lines were treated with different concentrations of H-4073 and cell proliferation was assessed by MTT assay. ***E:*** UM-SCC-74A cells were treated with different concentrations of H-4073 for 2 hours. STAT3, FAK, Akt and p38 phosphorylation was examined by Western blotting and equal protein loading was verified by stripping the blots and reprobing with GAPDH antibody.

The growth inhibitory effects of cisplatin in a panel of HNSCC cell lines were evaluated using MTT assay. As shown in Fig. 1A, cisplatin treatment inhibited the growth of HNSCC cell lines in a dose dependent manner. We selected two cell lines (CAL27-CisR and UM-SCC-74A) that were most resistant to cisplatin for further experiments. UM-SCC-74A is squamous cell carcinoma cell line derived from a base of the tongue tumor. This cell line was picked to mimic natural or inherent cisplatin resistance. CAL27-CisR was selected by growing the parental tongue squamous cell carcinoma cell line (CAL27) in increasing concentrations of cisplatin over an extended period of time [27]. This cell line was generated to mimic acquired cisplatin resistant phenotype. Curcumin has been extensive studied for its STAT3-inhibitory properties and anti-tumor activity [31]. However, due to its poor bioavailability and rapid metabolism, curcumin has not transitioned to clinic. Therefore, we have developed a synthetic analog of curcumin, H-4073 (Fig. 1B). In our study, H-4073 (10 µM) demonstrated >5-fold higher cellular uptake in a head and neck cancer cell line (UM-SCC-74A) as compared to curcumin (100 µM, Fig. 1 C). H-4073 was highly effective in inhibiting cell proliferation of all HNSCC cell lines that were tested, irrespective of their p53 status or human papillomavirus status (HPV, Figure 1D). In our mechanistic study, we observed a marked inhibition of STAT3, FAK and Akt phosphorylation by H-4073 treatment in a concentration-dependent manner (Fig. 1E). In addition, H-4073 treatment also induced the activation of p38 MAPK, a stress activated kinase that is known to mediate cell death.

### H-4073 synergistically enhanced the anti-tumor efficacy of cisplatin in HNSCC cells

**Figure 2:**
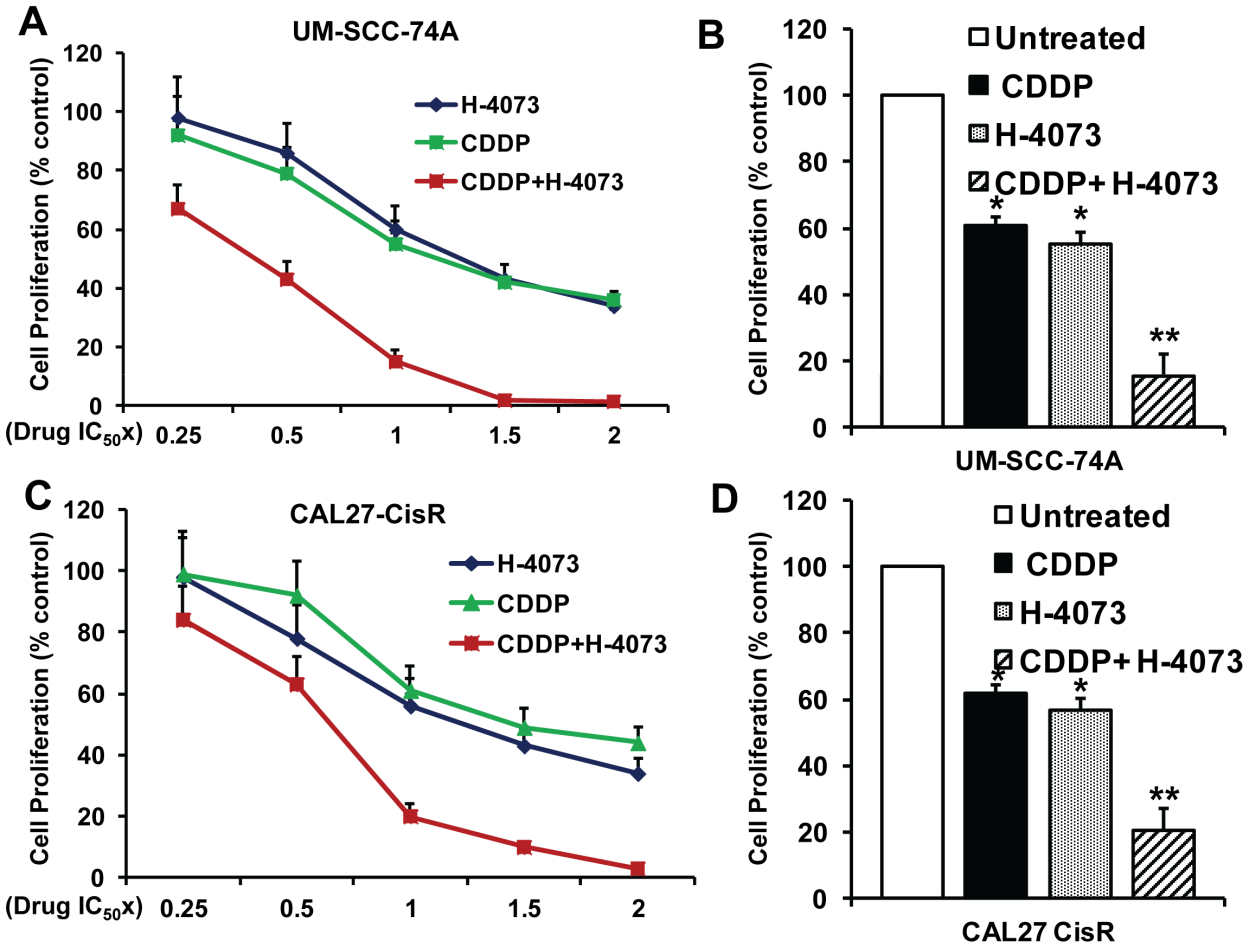
Synergistic anti-proliferative effects of H-4073 and cisplatin in head and neck cancer cells. ***A and C:*** UM-SCC-74A *(* ***A*** *)* or CAL27-CisR *(* ***C*** *)* cells were treated with H-4073 or cisplatin (CDDP) alone or in combination at 0.25, 0.5, 1, 1.5 and 2 times their respective IC_50_ doses and cell proliferation was assessed by MTT assay. Results were analyzed according to Chou-Talalay method and the combination index (CI) values calculated by CompuSyn software. ***B and D:*** Bar diagrams showing cell proliferation results for UM-SCC-74A *(* ***B*** *)* and CAL27-CisR *(* ***D*** *)* at IC_50_ doses of H-4073 and cisplatin (CDDP). *, represents a significant difference (p<0.05) as compared to no treatment group and **, represents a significant difference (p<0.05) as compared to single treatment groups.

We next examined if H-4073 treatment could reverse cisplatin resistance in head and neck cancer cells and enhance its anti-tumor efficacy in a synergistic manner. We selected two cisplatin-resistant cell lines, UM-SCC-74A (naturally cisplatin resistant) and CAL27-Cis-R (generated in our laboratory) for all our studies. The anti-proliferative effect of H-4073 and cisplatin (CDDP) combination treatment was measured by calculating the combination index (CI) according to Chou-Talalay method [28] using a fixed dose ratio. Both H-4073 and CDDP were added to the tumor cell cultures at 0.25×, 0.5×, 1×, 1.5× and 2× their respective IC_50_ doses. Cell proliferation in both the cell lines were markedly decreased following combination treatment at the multiple paired concentrations as compared to treatment with either of single agents alone (Fig. 2). Combination index (CI) for different effective doses (ED) was calculated using CompuSyn software. CI values for UM-SCC-74A cells at ED50, ED75 and ED90 were 0.550, 0.537 and 0.244 respectively. The CI values for CAL27-CisR cells were 0.628, 0.606 and 0.507 at ED50, ED75 and ED90, respectively. These results suggest that H-4073 and cisplatin combination treatment was highly effective in inhibiting tumor cell proliferation in a synergistic manner in both the cisplatin resistant cell lines.

**Figure 3:**
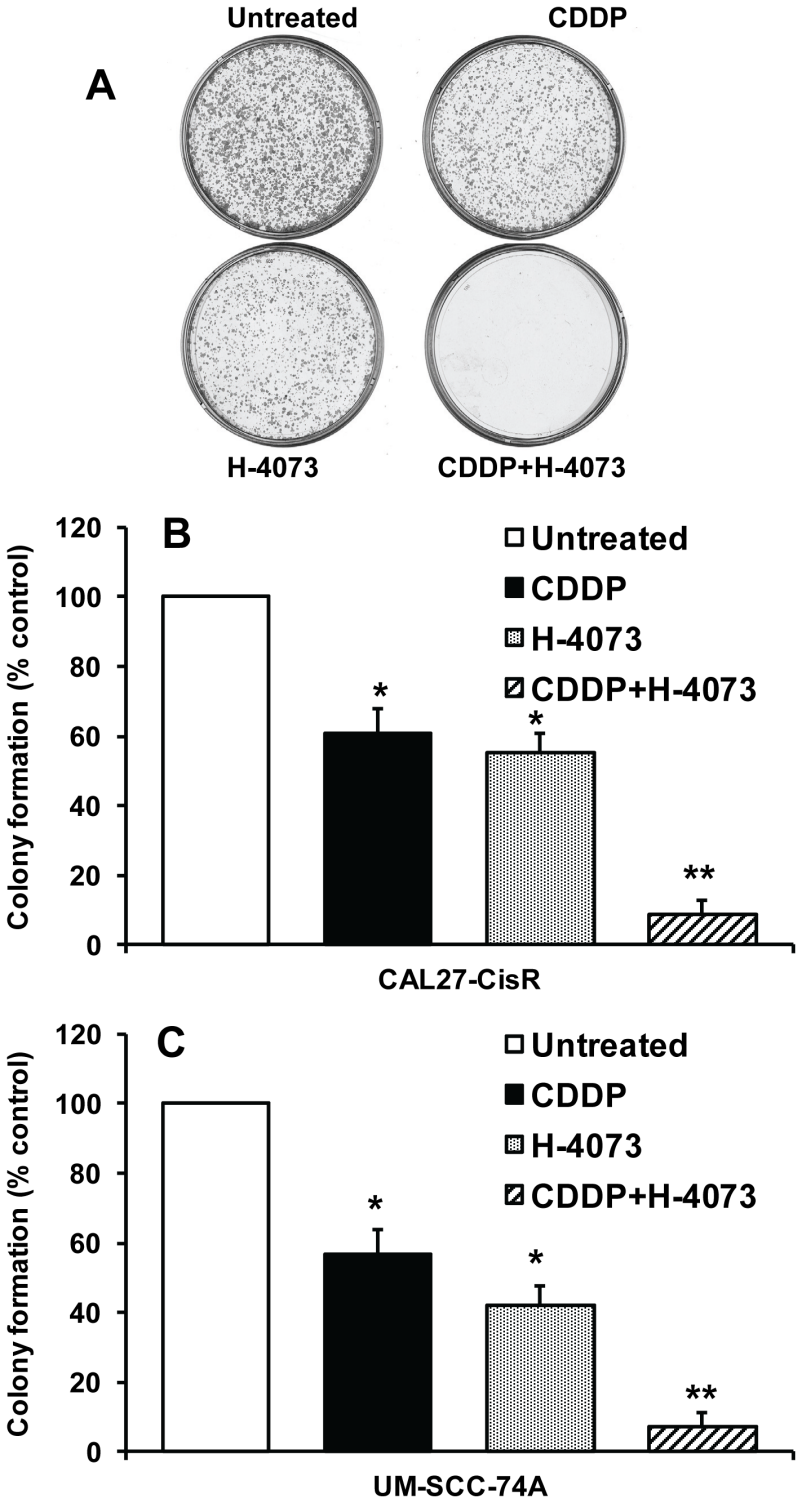
H-4073 markedly reduces tumor cell colony formation. CAL27-CisR *(* ***A–B*** *)* or UM-SCC-74a *(* ***C*** *)* cells were treated with H-4073 or cisplatin (CDDP) alone or in combination for 48 hours. Four thousand viable cells from each group were cultured for additional 10 days in 6-cm plates. Each assay was photographed and the number of colonies analyzed. *, represents a significant difference (p<0.05) as compared to no treatment group and **, represents a significant difference (p<0.05) as compared to single treatment groups.

We next investigated the effect of H-4073 alone or in combination with cisplatin on tumor cell colony formation. As observed with cell proliferation assay, a combination treatment with H-4073 and cisplatin at their respective IC_50_ doses inhibited tumor cell colony formation in a synergistic manner (93% in UM-SCC-74A and 92% in CAL27-CisR cells) (Fig. 3A–C).

### H-4073 inhibited cell migration and enhanced tumor cell apoptosis

**Figure 4:**
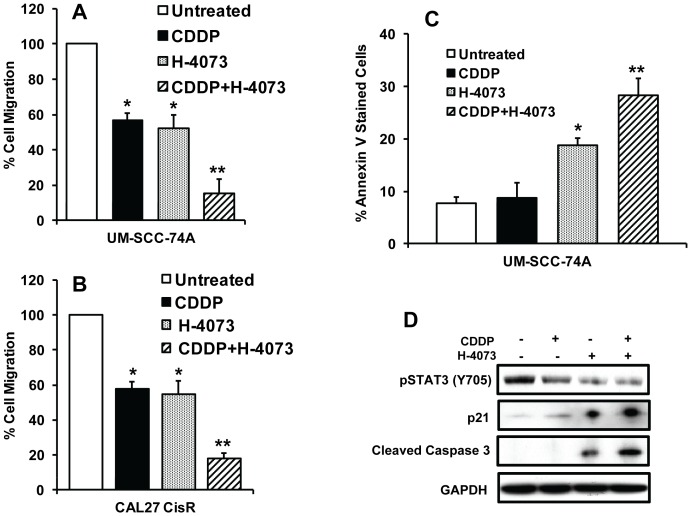
H-4073 and cisplatin significantly inhibits tumor cell migration and enhances apoptosis. ***A–B:*** Tumor cell motility was examined by scratch assay. Each assay was photographed and distances between the migrating cell edges were quantified and percentage cell migration was calculated. *, represents a significant difference (p<0.05) as compared to no treatment group and **, represents a significant difference (p<0.05) as compared to single treatment groups. ***C–D:*** UM-SCC-74A cells were treated with H-4073 or cisplatin (CDDP) alone or in combination. After 24 hours, cells were either stained with Annexin V and analyzed by flow cytometry or Western blotted for pSTAT3, p21 or cleaved caspase 3. Equal protein loading was verified by stripping the blots and reprobing with GAPDH antibody.

We next examined the effect of H-4073 and cisplatin combination treatment on tumor cell motility by scratch assay. H-4073 and cisplatin treatment alone showed 48% and 44% inhibition of UM-SCC-74A cell motility and 46% and 42% inhibition of CAL27-CisR cell motility, respectively (Fig. 4A–B). H-4073 when combined with cisplatin showed significantly higher inhibition of UM-SCC-74A and CAL27-CisR cell motility (85% and 82%), respectively. In next set of experiments, we examined if H-4073-mediated anti-tumor effects are mediated by apoptosis. Indeed, H-4073 in combination treatment with cisplatin showed significantly higher tumor cell apoptosis (Annexin V staining, Fig. 4C) as compared to untreated cells or H-4073 and cisplatin-treated cells alone. In addition, combination treatment significantly inhibited STAT3 activation and markedly increased the levels of activated caspase 3 and p21 (Fig. 4D).

### H-4073 significantly enhanced the therapeutic efficacy of cisplatin in cisplatin resistant head and neck cancers

**Figure 5:**
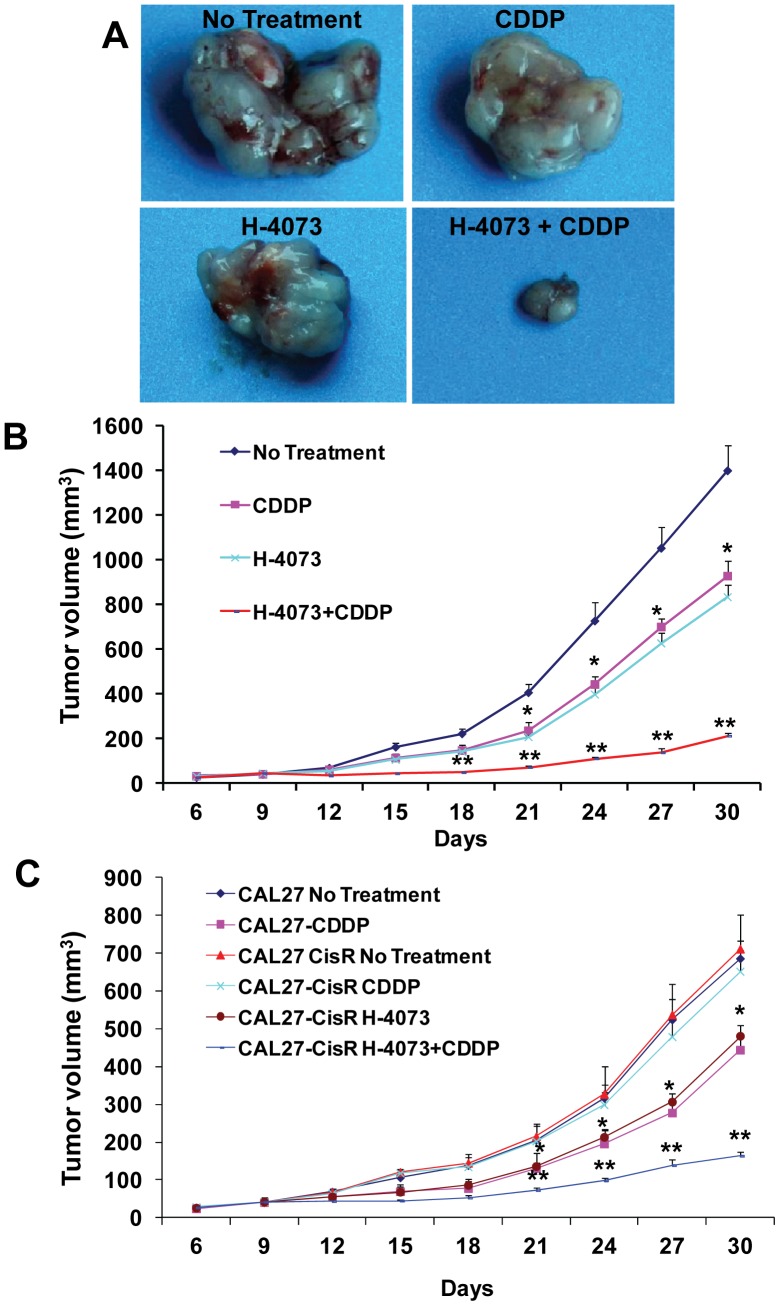
H-4073 and cisplatin combination treatment significantly inhibits tumor growth. Animals bearing UM-SCC-74A, CAL27 and CAL27-CisR were treated with H-4073 (50 ppm) or cisplatin (CDDP, 5 mg/kg) alone or in combination. ***A:*** Representative photomicrographs of UM-SCC-74A tumors from untreated, cisplatin (CDDP), H-4073, or CDDP and H-4073-treated groups. ***B:*** Tumor growth curves for UM-SCC-74A tumors treated with cisplatin (CDDP), H-4073, or CDDP and H-4073. ***C:*** Tumor growth curves for CAL27 and CAL27-CisR tumors treated with cisplatin (CDDP), H-4073, or CDDP and H-4073. *, represents a significant difference (p<0.05) as compared to no treatment group and **, represents a significant difference (p<0.05) as compared to single treatment groups.

Our *in vitro* data suggested that H-4073 significantly reversed cisplatin resistance in head and neck cancer cells. We further validated our *in vitro* results by using an athymic nude mouse xenograft model. In the first set of experiments, we used a naturally resistant head and neck cancer cell line (UM-SCC-74A). H-4073 (50 ppm) and cisplatin (5 mg/kg) treatment alone showed 33% and 39% tumor growth inhibition at day 30, respectively (Fig. 5A–B). H-4073 in combination with cisplatin showed significantly higher tumor growth inhibition as compared to untreated group (84%) or single agent alone (Fig. 5A–B). In addition, the combination treatment was very well tolerated, and it did not cause any animal toxicity or induce significant decrease in body weight.

In the second set of experiments, we used cisplatin-resistant cell line (CAL27-CisR, IC_50_ 28 µmol/L) and its parental cisplatin-sensitive cell line (CAL27, IC_50_ 3 µmol/L). As we have previously demonstrated [27], cisplatin treatment (5 mg/kg) of animals bearing CAL27-CisR tumors did not significantly affect tumor growth (9%) at day 30, whereas cisplatin treatment of its parental cells (CAL27) markedly decreased tumor growth (45%). H-4073 treatment in CAL27-CisR cells was significantly more effective in reducing tumor burden (32%). H-4073 and cisplatin combination treatment was most effective in inhibiting tumor growth of CAL27-CisR tumors (77%, Fig. 5C).

### H-4073 and cisplatin combination treatment significantly inhibited STAT3 phosphorylation *in vivo* and enhanced tumor cell apoptosis

**Figure 6:**
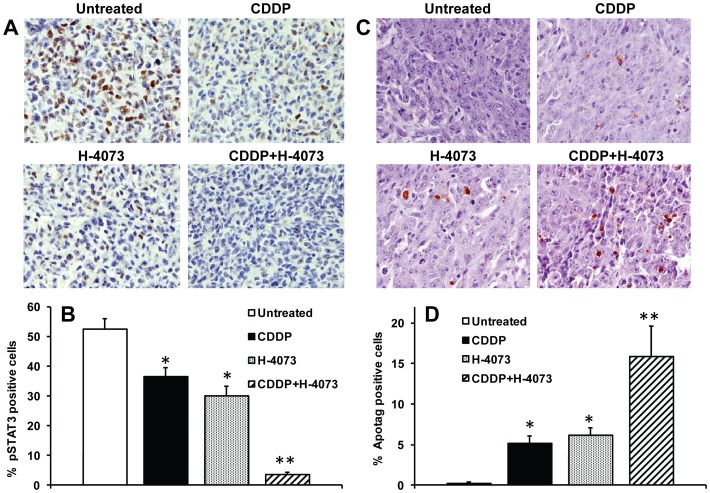
H-4073 and cisplatin treatment significantly inhibits STAT3 phosphorylation and markedly increases tumor cell apoptosis, *in vivo*. ***A–D:*** Paraffin-embedded UM-SCC-74A tumor samples were stained for pSTAT3 (Y705) and apoptotic cells (Apop Tag kit). ***A:*** Representative photomicrographs of tumor samples stained for pSTAT3 from untreated, cisplatin (CDDP) or H-4073 alone or combination groups. ***B:*** pSTAT3 positive cells were quantified in 5 high power fields (400×) of each tumor samples and percentage of positive cells calculated. ***C:*** Representative photomicrographs of tumor samples stained for apoptotic cells from untreated, cisplatin (CDDP) or H-4073 alone or combination groups. ***D:*** TUNEL-positive cells were quantified in 5 high power fields (400×) of each tumor samples and percentage of positive cells calculated. *, represents a significant difference (p<0.05) as compared to no treatment group and **, represents a significant difference (p<0.05) as compared to single treatment groups.

In our *in vitro* study, we have observed that H-4073 is a potent inhibitor of STAT3 phosphorylation. We next examined if H-4073 treatment inhibited SAT3 phosphorylation *in vivo*. UM-SCC-74A tumors from mouse xenograft model were stained with pSTAT3 antibody. H-4073 and cisplatin treatment significantly inhibited STAT3 phosphorylation (43% and 30%, respectively). H-4073 and cisplatin combination treatment was most effective in inhibiting STAT3 phosphorylation (94%) (Fig. 6A–B). Similarly, H-4073 and cisplatin combination treatment was most effective in inducing apoptosis in tumor cells (Fig. 6C–D).

### H-4073 inhibited tumor angiogenesis by downregulating VEGF secretion from tumor cells

**Figure 7:**
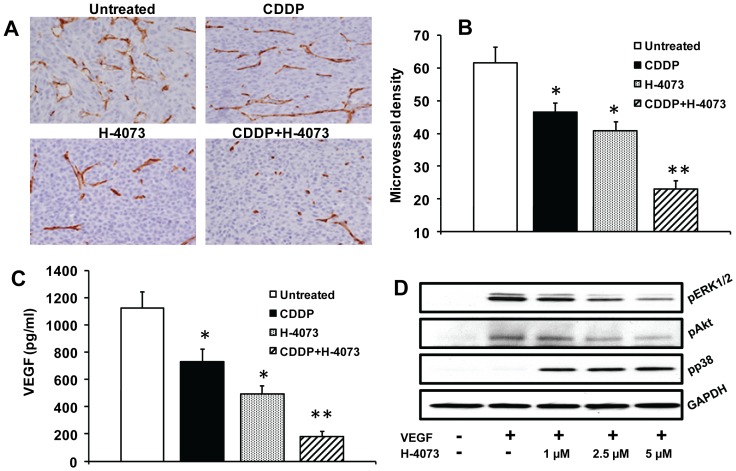
H-4073 and cisplatin combination treatment significantly inhibits tumor angiogenesis. ***A:*** Representative photomicrographs of tumor blood vessel staining for untreated, cisplatin (CDDP) or H-4073 alone or combination groups for UM-SCC-74A tumors. ***B:*** Microvessel density in the tumor samples was calculated by counting 5 random fields (200×) and expressed as vessel density ± SE. ***C:*** UM-SCC-74A cells were treated with cisplatin or H-4073 alone or in combination. After 72 hours, culture supernatants were collected and assayed for VEGF levels. *, represents a significant difference (p<0.05) as compared to no treatment group and **, represents a significant difference (p<0.05) as compared to single treatment groups. ***D:*** Endothelial cells were treated with VEGF in the presence or absence of different concentrations of H-4073 for 30 minutes. ERK1/2, Akt and p38 phosphorylation was examined by Western blotting and equal protein loading was verified by stripping the blots and reprobing with GAPDH antibody.

A number of studies have shown that STAT3 is a key regulator of angiogenesis [16], [32]. We next examined if H-4073 and cisplatin combination treatment affected tumor angiogenesis. H-4073 and cisplatin treatment alone showed 24% and 31% inhibition of tumor angiogenesis in UM-SCC-74A (Fig. 7A–B), whereas H-4073 and cisplatin combination treatment showed 62% inhibition of tumor angiogenesis. We next examined if H-4073 inhibited tumor angiogenesis by blocking VEGF production by tumor cells. UM-SCC-74A cells were treated with H-4073 and VEGF levels in culture supernatants were measured by ELISA. Untreated UM-SCC-74A cells produced high levels of VEGF (1121 pg/ml/10^6^ cells, Fig. 7C). H-4073 and cisplatin treatment alone showed 36% and 55% inhibition of VEGF levels. H-4073 and cisplatin combination treatment significantly inhibited VEGF production (83%). In the next set of experiments, we examined if H-4073 could directly affect angiogenic function of endothelial cells by inhibiting VEGF signaling. Our results from this study demonstrate that H-4073 markedly inhibit VEGF-mediated ERK1/2 and Akt phosphorylation (Fig. 7D). In addition to inhibiting the pro-survival signaling molecules (ERK1/2 and Akt) H-4073 also activated p38 MAPK (a pro-apoptotic molecule) in a dose dependent manner (Fig. 7D).

## Discussion

Patients with head and neck cancer encompass a heterogeneous group and even with advancement in treatment options, the overall survival rate for patients with advanced disease has not changed substantially over recent decades [33]. Surgery followed by adjuvant radiotherapy has long been used in the management of patients with HNSCC [34]. More recently, platinum-based regimens are being integrated into the treatment options [35]. However, many of the patients develop resistance to cisplatin, one of the most widely used platinum agent, leading to treatment failure [21], [36]. Therefore, there is an urgent need to develop novel therapeutic agents that specifically targets pro-survival pathways. Recent studies have shown that STAT3 is constitutively activated in tumor cells and is overexpressed in cisplatin-resistant cell lines [21], [37]. STAT3 is involved in the suppression of apoptosis in cisplatin resistant cell lines by upregulating survival genes like Bcl-xL, Bcl-2 and survivin [38], [39]. Therefore, we hypothesized that targeting STAT3 could reverse the resistant phenotype in tumor cells, thereby enhancing the anti-tumor effects of cisplatin treatment in HNSCC.

To test this hypothesis, we have carried out combination treatment studies *in vitro* and *in vivo* using athymic nude xenograft model. In this study, we selected a novel analog of curcumin (H-4073) because curcumin has been shown to be a potent inhibitor of STAT3 and demonstrate anti-tumor activity in various cancers including head and neck cancer. However, its instability *in vitro* and poor bioavailability has limited its clinical application. We therefore developed a novel DAP analog of curcumin (H-4073) [24]. In our bioabsorption studies, H-4073 demonstrated significantly higher (>5 fold) cellular uptake in head and neck cancer cells as compared to curcumin. We did note that the level of bioabsorption of H-4073 varied between different cell lines. We think that this difference could be due to differential expression of multiple drug influx and efflux transporters in these cell lines [40]. We then used various HNSCC cell lines (HPV positive, HPV negative, wild-type p53 or mutant p53) to check the ability of H-4073 to inhibit growth *in vitro*. We found that H-4073 inhibited the growth of cell lines irrespective of p53 or HPV status in a dose dependent manner. If this finding could be broadly translated into clinical efficacy in heterogeneous HNSCC tumors (50–80% mutant p53, 60–80% of oropharynx tumors are HPV positive), H-4073 could represent a promising new agent for targeted therapy in HNSCC. Interestingly, majority of the head and neck cancer patients with HPV positive tumors respond very well to traditional chemotherapy with cisplatin and demonstrate significantly favorable clinical outcome [33], [41]. It is the patients with HPV negative tumors that show markedly poor clinical outcome and often develop resistance to chemotherapy. Therefore, this non-HPV associated patient population could tremendously benefit from the addition of targeted therapies to currently used treatment regimens. In this study, we have used 2 HPV-negative HNSCC cell lines (UM-SCC-74A and CAL27-CisR). UM-SCC-74A cell line is derived from a head and neck cancer patient with base of tongue tumor and is highly resistant to both chemotherapy and radiation treatment [42], [43]. In addition, we generated a cisplatin-resistant cell line (CAL27-CisR, IC_50_ 28 µM) in our laboratory by culturing a cisplatin sensitive tongue SCC cell line CAL27 (IC_50_ 3 µM) in increasing doses of cisplatin over a period of time [27].

H-4073 treatment was very effective in inhibiting tumor cell proliferation, colony formation and cell migration. In addition, H-4073 pretreatment significantly reversed cisplatin resistance in both naturally resistant head and neck cancer cell line (UM-SCC-74A) as well as in cell line with acquired cisplatin resistance (CAL27-CisR). Combination index analysis by Chou-Talalay method [28] demonstrated that H-4073 and cisplatin combination treatment is highly effective and synergistic in mediating anti-tumor effects. These potent anti-tumor effects of H-4073 could be due to its inhibitory effects on a number of key signaling pathways including STAT, FAK, Akt and VEGF. STAT3 activation has been demonstrated in HNSCC tumor samples obtained from patients that developed resistance to cetuximab or cisplatin treatment [21], [44]. Huang et al have also demonstrated that inhibition of STAT3 signaling in cisplatin-resistant gastric cancer cells by using siRNA significantly reversed cisplatin resistance [45]. We also observed a marked decrease in Akt phosphorylation in H-4073 treated cells. Akt inhibition by H-4073 could also be contributing to reversal of chemoresistance in head and neck cancer cells as Akt has been shown to mediate chemoresistance in a number cancer types [46], [47]. A number of studies have demonstrated the role of FAK in cell survival particularly anchorage dependent survival [48], [49]. Recent studies have also highlighted the role of FAK in cell migration [50]. We observed that H-4073 treatment markedly inhibited FAK phosphorylation. This could be a direct effect of H-4073 on FAK phosphorylation or indirectly mediated through STAT3 as we have previously shown that STAT3 can regulate FAK activation in head and neck cancer cells [29].

To further determine if the observed *in vitro* synergy between H-4073 and cisplatin extends to the *in vivo* setting, we used a nude mouse xenograft model to study the effect of combination treatment on tumor growth and tumor angiogenesis. Indeed combination treatment of H-4073 (50 ppm) and cisplatin (5 mg/kg) induced significant reduction in tumor burden. This marked inhibition of tumor growth by combination treatment could be because of H-4073-mediated inhibition of STAT3, Akt and FAK signaling pathways as well as reduction in the formation of new blood vessels by reducing VEGF production and inhibiting VEGF signaling. H-4073 and cisplatin combination treatment was very well tolerated in the animals. It did not cause any animal mortality or induced any significant weight loss or induced any major systemic toxicity such as dry scaly skin or respiratory distress that has been reported in animals treated with chemotherapy treatment with other small molecular weight inhibitors [51]. In conclusion, we have shown that H-4073 significantly enhances the anti-tumor effects of cisplatin treatment by inhibiting tumor growth and tumor angiogenesis. Therefore, H-4073 with its low toxicity and good bioavailability is a potentially novel candidate to enhance the therapeutic efficacy of cisplatin treatment in head and neck cancer patients.
